# Transcriptional profiling reveals molecular basis and novel genetic targets for improved resistance to multiple fermentation inhibitors in *Saccharomyces cerevisiae*

**DOI:** 10.1186/s13068-015-0418-5

**Published:** 2016-01-13

**Authors:** Yingying Chen, Jiayuan Sheng, Tao Jiang, Joseph Stevens, Xueyang Feng, Na Wei

**Affiliations:** Department of Civil and Environmental Engineering and Earth Sciences, University of Notre Dame, 106E Cushing Hall of Engineering, Notre Dame, South Bend, IN 46556 USA; Department of Biological Systems Engineering, Virginia Polytechnic Institute and State University, Blacksburg, VA USA; Department of Civil and Environmental Engineering, University of Pittsburgh, Pittsburgh, PA USA

**Keywords:** Yeast, Acetic acid, Furfural, RNA-seq, Transcription factors, Metabolic engineering

## Abstract

**Background:**

Lignocellulosic biomass is a promising source of renewable biofuels. However, pretreatment of lignocellulosic biomass generates fermentation inhibitors that adversely affect the growth of industrial microorganisms such as *Saccharomyces cerevisiae* and prevent economic production of lignocellulosic biofuels. A critical challenge on developing *S. cerevisiae* with improved inhibitor resistance lies in incomplete understanding of molecular basis for inhibitor stress response and limited information on effective genetic targets for increasing yeast resistance to mixed fermentation inhibitors. In this study, we applied comparative transcriptomic analysis to determine the molecular basis for acetic acid and/or furfural resistance in *S. cerevisiae*.

**Results:**

We recently developed a yeast strain YC1 with superior resistance to acetic acid, furfural, and their mixture through inverse metabolic engineering. In this study, we first determined transcriptional changes through RNA sequencing in YC1 versus the wild-type strain S-C1 under three different inhibitor conditions, including acetic acid alone, furfural alone, and mixture of acetic acid and furfural. The genes associated with stress responses of *S. cerevisiae* to single and mixed inhibitors were revealed. Specifically, we identified 184 consensus genes that were differentially regulated in response to the distinct inhibitor resistance between YC1 and S-C1. Bioinformatic analysis next revealed key transcription factors (TFs) that regulate these consensus genes. The top TFs identified, Sfp1p and Ace2p, were experimentally tested as overexpression targets for strain optimization. Overexpression of the *SFP1* gene improved specific ethanol productivity by nearly four times, while overexpression of the *ACE2* gene enhanced the rate by three times in the presence of acetic acid and furfural. Overexpression of *SFP1* gene in the resistant strain YC1 further resulted in 42 % increase in ethanol productivity in the presence of acetic acid and furfural, suggesting the effect of Sfp1p in optimizing the yeast strain for improved tolerance to mixed fermentation inhibitor.

**Conclusions:**

Transcriptional regulation underlying yeast resistance to acetic acid and furfural was determined. Two transcription factors, Sfp1p and Ace2p, were uncovered for the first time for their functions in improving yeast resistance to mixed fermentation inhibitors. The study demonstrated an omics-guided metabolic engineering framework, which could be developed as a promising strategy to improve complex microbial phenotypes.

**Electronic supplementary material:**

The online version of this article (doi:10.1186/s13068-015-0418-5) contains supplementary material, which is available to authorized users.

## Background

Lignocellulosic biomass has the potential to contribute substantially to future global energy demands, because it is low in cost, is available at large-scale, does not compete with food production, and has high potential to reduce greenhouse gas emission [1–4]. However, inefficient conversion of solubilized plant cell wall materials into biofuels has hindered commercial scale processes. Lignocellulosic biomass materials need to undergo harsh (physico)chemical treatment designed to release sugar compounds [5, 6], but at the same time, the hydrolysis pretreatment generates toxic byproducts such as weak acids, furan aldehydes, and phenolic compounds (referred to as “fermentation inhibitors”) [7–9]. A robust inhibitor resistance fermenting microorganism is critically important for developing economically viable lignocellulosic biofuels, but this remains a major technical barrier [9].

Two major groups of fermentation inhibitors generated from pretreatment of lignocellulosic biomass are weak acids (e.g., acetic acid and formic acid) and furan aldehydes [e.g., furfural and 5-hydroxy methylfurfural (HMF)] [9, 10]. Particularly, since hemicellulose in the plant cell wall is ubiquitously acetylated [11, 12], typical acidic pretreatment of lignocellulosic biomass generates substantial amounts of acetic acid as an unavoidable fermentation inhibitor in hydrolysates [7, 13] and acetic acid is usually of the highest concentration among fermentation inhibitors in cellulosic hydrolysates [7, 14–18]. Furfural and HMF are major byproducts generated from hydrolysis and dehydration of pentose and hexose sugars [9, 10].

The yeast *Saccharomyces cerevisiae* is a preferred and widely used platform microorganism in industrial fermentation, but the toxic nature of cellulosic hydrolysates and low tolerance of the microorganism prevent efficient bioethanol production from cellulosic sugars [19, 20]. Uptake of weak acids decreases intracellular pH, which triggers the action of the plasma membrane ATPase to pump protons out of the cell at the expenses of ATP hydrolysis [21–24]. In addition, weak acids also cause intracellular anion accumulation, which interferes with enzymatic reactions and causes toxicity [25, 26]. Furan aldehydes inhibit enzymes of central carbon metabolism [27–29] and energy metabolism [30], and cause depletion of NAD(P)H pools and oxidative stress [10, 31–33]. The key challenge of engineering inhibitor-resistant yeast lies in that the resistance phenotype usually involves complex multi-genic regulations among disparate stress responses.

There have been significant advances in determining inhibitor stress response mechanisms for improving yeast resistance to individual fermentation inhibitors [9, 34]. For example, resistance to furan aldehydes could be enhanced by overexpressing genes related to aldehyde reduction [35, 36], spermidine synthesis [37], pentose phosphate pathway [38, 39], or multidrug resistance and stress responses [9, 40]. As for tolerance to weak acids such as acetic acid, analysis of transcriptional response of *S. cerevisiae* to acetic acid stress showed up-regulation of various genes involved in glycolysis, the Krebs cycle and ATP synthesis [41–43] and the important role of the transcription factor Haa1p in regulating the cell-wide transcriptional adaptation to acetic acid in yeast [42, 44, 45]. Genetic targets related to resistance to individual fermentation inhibitors in *S. cerevisiae* were reported in some previous studies [46, 47]. For example, earlier studies found that overexpression of Msn2p [46] and Yap1p [48] could improve furfural resistance in the yeast. While prior studies are mostly focused on characterization of genetic mechanisms for yeast stress response to individual inhibitory compounds, cellulosic hydrolysates contain mixed fermentation inhibitors with distinct toxicity mechanisms rather than a single inhibitor. Some recent works reported improved yeast resistance to cellulosic hydrolysates through evolutionary engineering [49–51], and systematic analysis was used in previous studies to understand molecular basis for yeast inhibitor resistance [51–56]. It was found that different mechanisms could be adopted by the yeast to resist hydrolysates inhibitors (e.g. acetic acid, furfural, and HMF) [51]. However, there is still limited information on what genetic perturbation targets could be elicited to improve yeast resistance to mixed fermentation inhibitors. Therefore, a better understanding of genetic regulatory networks underlying the resistance to mixed fermentation inhibitors in *S. cerevisiae* is needed to develop strains with enhanced tolerance to cellulosic hydrolysates.

We recently developed a yeast strain that has superior inhibitor resistance through inverse metabolic engineering [57]. In the present study, we performed comparative transcriptomic analysis using RNA deep sequencing (RNA-seq) to determine transcriptional response in *S. cerevisiae* to acetic acid and/or furfural, and to identify key transcription factors (TFs) that regulate tolerance to mixed inhibitors in the yeast. First, the genome-wide transcriptional changes in the resistant strain versus the wild-type control strain were identified by transcriptomic analysis under three different inhibitor conditions, including acetic acid alone, furfural alone, and mixture of acetic acid and furfural. Then, the TFs that regulate the core genes with significant changes in expression under stress of both inhibitors were identified and top TFs were tested experimentally as overexpression targets for strain optimization. Our results advance fundamental understanding of the genetic regulatory mechanisms underlying yeast resistance to major fermentation inhibitors in cellulosic hydrolysates. We also report novel transcription factors involved in regulating resistance to mixed inhibitor stress. The transcriptome-guided metabolic engineering demonstrated here could be a promising strategy to improve complex phenotypes in yeast, particularly in the cases where coordinated reprogramming of a number of genes is needed.

## Materials

### Strains and plasmids

All the strains and plasmids used in this study are summarized in Table 1. The *S. cerevisiae* strains SR8, SR8-*trp*, and SR8-*4* were kindly provided by Dr. Yong-Su Jin’s lab. The strain SR8-*trp* was transformed with yeast genomic DNA library on a multicopy plasmid pRS424 and a yeast transformant YC1 with improved resistance to acetic acid, and furfural was obtained through the inverse metabolic engineering approach in our recent work [57]. The *Escherichia coli* TOP10 strain was used for gene cloning and manipulation.

**Table 1:** Plasmids and Strains

Plasmids and strains	Description	References
Plasmids		
pRS424	*TRP1*, a multicopy plasmid	[76]
pRS425	*LEU2*, a multicopy plasmid	[76]
pRS424-*WHI2*	pRS424 with insert of S288c yeast genomic DNA fragment chrXV: 409,259-412,369 (containing complete sequence of the *WHI2* gene)	This study
pRS424GPD	pRS424 with GPD promoter	[76]
pRS425GPD	pRS425 with GPD promoter	[76]
pRS424GPD-*WHI2*	*WHI2* expressed in pRS424GPD	This study
pRS424GPD-*SFP1*	*SFP1* expressed in pRS424GPD	This study
pRS424GPD-*ACE2*	*ACE2* expressed in pRS424GPD	This study
pRS425GPD-*SFP1*	*SFP1* expressed in pRS425GPD	This study
pRS425GPD-*ACE2*	*ACE2* expressed in Prs425GPD	This study
Strains		
D452-2	*MATa, leu2, his3, ura3, can1*	[77]
SR8	D452-2 expressing *XYL1, XYL2,* and *XKS1* through integration, evolutionary engineering in xylose-containing media, and *ALD6* deletion	[40]
SR8-*trp*	SR8 with *TRP1* disrupted	Developed in Dr. Yong-Su Jin lab
SR8-*4*	SR8 with *TRP1*, *LEU2, HIS3 and URA3* disrupted	Developed in Dr. Yong-Su Jin lab
S-C1	SR8-*trp* harboring pRS424GPD, as a control	This study
YC1	SR8-*trp* harboring pRS424-*WHI2*	This study
S- *SFP1*	SR8-*trp* harboring pRS424GPD- *SFP1*	This study
S-*ACE2*	SR8-*trp* harboring pRS424GPD- *ACE2*	This study
S-C2	SR8-*4* harboring pRS424GPD and pRS425GPD, as a control	This study
S-*WHI2*-c	SR8-*4* harboring pRS424GPD-*WHI2* and pRS425GPD	This study
S-*WHI2*-*SFP1*	SR8-*4* harboring pRS424GPD- *WHI2* and pRS425GPD- *SFP1*	This study

The *TDH3* promoter is often referred to as the *GPD* promoter, which is used in the pRS4XX series of expression vectors [76]

*GPD* stands for Glyceraldehyde-3-phosphate dehydrogenase, encoded by the *TDH3* gene

### Enzymes, primers, and chemicals

Restriction enzymes, DNA-modifying enzymes, and PCR reagents were purchased from New England Biolabs (Beverly, MA, USA). The reaction conditions were set-up following the manufacturer’s instructions. All general chemicals and media components were purchased from Sigma-Aldrich (St. Louis, MO, USA) or Fisher Scientific (Pittsburgh, PA, USA). Primers for both PCR and sequencing were synthesized by Integrated DNA Technologies (Coralville, IA, USA) and are listed in Table 2.

**Table 2:** Primers used in this study

Target	Primer sequence
*WHI2*	Forward GCCGGATCCAAAAATGGACGATATAATCACGCAAG
	Reverse GCCGTCGACTCACTGCACCCCAATAACGC
*SFP1*	Forward GCCCCCGGGATGGATTTTACAACAATGACTATG
	Reverse GCCGTCGACTTAGTGAGTGGAGTGGCCCC
*ACE2*	Forward GCCACTAGTATGGATAACGTTGTAGATCCGTG
	Reverse GCCGTCGACTCAGAGAGCATCAGTTTCGTTTG
T3 promoter	AATTAACCCTCACTAAAGGG
T7 promoter	TAATACGACTCACTATAGGG

### Plasmid and strain construction

To construct overexpression plasmids with different identified transcriptional factors, the complete open reading frames of transcriptional factors were amplified by PCR with the primers listed in Table 2. The PCR products were subsequently digested and ligated to appropriate multiple cloning sites of the plasmid pRS424GPD or pSR425GPD. The overexpression vectors were transformed, respectively, to the strain SR8-*trp* using Yeast EZ-transformation Kit (BIO 101).

### Media and culture conditions

*Escherichia coli* strains were grown in Luria–Bertani medium at 37 °C, and 100 μg/mL of ampicillin was added to the medium when required. Yeast strains were routinely cultivated at 30 °C in YP medium (10 g/L of yeast extract and 20 g/L of peptone) or synthetic complete (SC) medium (6.7 g/L of yeast nitrogen base, 0.6 g/L complete supplement mixture) containing 20 g/L of d-glucose. SC media containing 20 g/L agar and glucose, 20 mg/L histidine and uracil without tryptophan and/or leucine (100 mg/L if needed) amendment was used to select transformants using *TRP1* and/or *LEU2* as auxotrophic markers.

### Batch fermentation experiments

Yeast cells were pre-cultured in SC medium containing 20 g/L glucose until stationary phase, and then were centrifuged, washed by sterilized water, and then inoculated into fermentation media containing glucose (20 g/L), acetic acid (2 g/L), and/or furfural (1 g/L). Batch fermentation experiments under oxygen-limited conditions were conducted in 125 mL non-baffled Erlenmeyer flasks containing 20 mL media at 30 °C and 100 rpm. The initial cell densities were adjusted to OD_600_ = 1 or 0.2. The initial pH of the media was adjusted to 4.0. Culture samples were taken from fermentation experiments to measure the OD_600_ and concentrations of metabolites. All fermentation experiments were set-up in biological duplicate.

### Sample preparation for RNA sequencing

Yeast cells were grown to early exponential phase under oxygen-limited conditions in 50 mL SC + glucose medium in 250 mL Erlenmeyer flasks in biological triplicate, and were exposed to different inhibitors for 4 hours before cell samples were collected for RNA-seq analysis. Four inhibitor conditions were applied for comparative transcriptomics study: (1) acetic acid (2 g/L), (2) furfural (1 g/L), (3) acetic acid (2 g/L) + furfural (1 g/L), and (4) blank control without any inhibitor. Cell samples taken from each replicate incubations were collected in pre-chilled Falcon tubes and were centrifuged at 4 °C for 1 min. The cell pellets were flash-frozen in liquid nitrogen and stored in −80 °C before analysis. Total RNA was extracted by PureLink RNA Mini Kitfrom Life Technology (Grand Island, NY) according to the supplier’s instructions. The RNA samples were then sent to Virginia Bioinformatics Institute for further quality and quantity evaluation, cDNA library preparation, and sequencing.

### Analytical methods

Fermentation metabolites including glucose, glycerol, acetic acid, and ethanol were quantified by high performance liquid chromatography (Agilent Technologies 1200 series) equipped with a refractive index detector and a Rezex ROA-Organic Acid H^+^ (8 %) column (Phenomenex Inc., Torrance, CA, USA). The column was eluted with 0.005 N H_2_SO_4_ as the mobile phase under the flow rate of 0.6 mL/min at 50 °C. Cell growth was monitored by measuring optical density at 600 nm (OD_600_) using UV–visible spectrophotometer (Thermo Fisher Scientific Inc., Waltham, MA, USA).

### RNA-seq and bioinformatics analysis

All the library preps were performed on Apollo 324 Robot (WaferGen, Fremont, CA, USA). Quality of total RNA was checked on Agilent BioAnalyzer 2100 (Agilent Technologies, Santa Clara, CA, USA). 500 ng of total RNA was enriched for polyA RNA using PrepXPolyA mRNA Isolation Kit (P/N 400047, WaferGen, Fremont, CA, USA). PolyA RNA was then converted into a library of template molecules using PrepX RNA-Seq for Illumina Library Kit, 24 samples (P/N 400046, WaferGen, Fremont, CA, USA) for subsequent cluster generation and sequencing by Illumina HiSeq. Briefly, polyA mRNA was fragmented into smaller pieces (~140nt). 3′ and 5′ adapters were ligated to the cleaved RNA fragments and converted to first-strand cDNA using reverse transcriptase, followed by second-strand synthesis. The products were then purified and enriched with 12 cycles of PCR to create the final cDNA library. The 280–300 bp libraries (160–180 bp insert) generated were validated using Agilent 2100 Bioanalyzer and quantitated using Quant-iT dsDNA HS Kit (Invitrogen) and qPCR. 12 indexed cDNA libraries were pooled and sequenced on each lane of HiSeq Rapid Run flow cell to generate 130–150 million single reads. Libraries were clustered onto a flow cell using Illumina’s TruSeq Rapid SR Cluster Kit 2500 (GD-402-4001) on the cBot, and sequenced 1 × 101 SR cycles using two TruSeq Rapid SBS Kit (50-cycles) (FC-402-4002).

Following sequencing, data were trimmed for both adaptor and quality using a combination of ea-utils [58] and Btrim [59]. Sequencing reads were then aligned to the genome using Tophat2/Bowtie2 [60] and counted via HTSeq [61]. QC summary statistics were examined to identify any problematic samples (e.g., total read counts, quality and base composition profiles (±trimming)), raw fastq formatted data files, aligned files (bam and text file containing sample alignment statistics), and count files (HTSeq text files). Following successful alignment, miRNA and mRNA differential expressions were determined and tested for significance using the Benjamini-Hochberg-corrected Wald Test in the R-package DESeq2 [62]. The gene ontology analysis was performed by using generic GO term mapper developed by Princeton University (http://go.princeton.edu/cgi-bin/GOTermMapper).

### Quantitative PCR (qPCR) validation

To validate the differentiated gene expressions from RNA-seq analysis, 24 samples analyzed by RNA-Seq were validated by TaqMan qPCR for differential expression of 3 target genes (YML038C-SC04151537, YHR127 W-SC04130738, and YJR096 W-SC04138893) and an endogenous control, YNL219C-SC04159779 (ALG9). 1.5 µg of total RNA was reverse transcribed using SuperScript VILO MasterMix kit (P/N 100012386, Invitrogen). 25 ng of cDNA was used for each TaqMan PCR reaction. Each sample with each target gene was done in triplicate. qPCR was performed using TaqMan Fast Advanced MasterMix (P/N4444557, Applied Biosystems), on ViiA7 instrument (Applied Biosystems) at 50 °C for 2 min, 95 °C for 20 s and 40 cycles at 95 °C 1 s and 60 °C for 30 s. Data were analyzed according to the ΔΔC_t_ method as described in the Invitrogen RT-PCR manual. The expression levels of all the target genes in qPCR analysis were found to be well correlated to those in RNA-seq analysis (Additional file 1: Figure S1).

### Transcription factor analysis

To identify the transcription factors that are most likely involved in regulating yeast transcriptomics, transcription factor analysis was accomplished using a previously published method [63]. In general, the YEASTRACT database (http://www.yeastract.com/) was solicited, in which the differentially expressed genes identified by the RNA-seq analysis were searched against all of the transcription factors (TFs) in the YEASTRACT database (only documented regulations with direct or indirect evidences were taken into consideration). To provide the TF profiles, the number of genes that a TF can regulate in the pool of genes that were found to be differentially expressed was calculated by YEASTRACT database. Then, it is divided by the total number of genes that were found to be differentially expressed.

## Results

### Fermentation under the stress of acetic acid and furfural

We have developed an inhibitor-resistant strain YC1 (Table 1) through our prior work using a genomic library-based inverse metabolic engineering approach [57]. Briefly, a genome-wide plasmid library was introduced into a parent *S. cerevisiae* strain, and the transformants were characterized and screened under stress conditions to identify the target of gene perturbation eliciting improved inhibitor resistance. The strain YC1 contains a multicopy plasmid with a yeast genomic DNA fragment insert (chrXV: 409,259–412,369). The insert harbors complete sequence of the gene *WHI2,* which encodes a cytoplasmatic globular scaffold protein required for activation of general stress response [64, 65]. Compared to the control strain S-C1 containing plasmid without the insert, the strain YC1 had significantly higher resistance to acetic acid [57]. The strain also showed significantly improved fermentation performance in corn stover hydrolysates compared to the control strain [57], suggesting its ability to resist mixed fermentation inhibitors. With the motivation to understand and compare transcriptional regulations under individual inhibitor stress versus mixed inhibitor stress in *S. cerevisiae*, we designed comparative transcriptomics experiments by focusing on two major inhibitors: acetic acid and furfural.

**Fig. 1:**
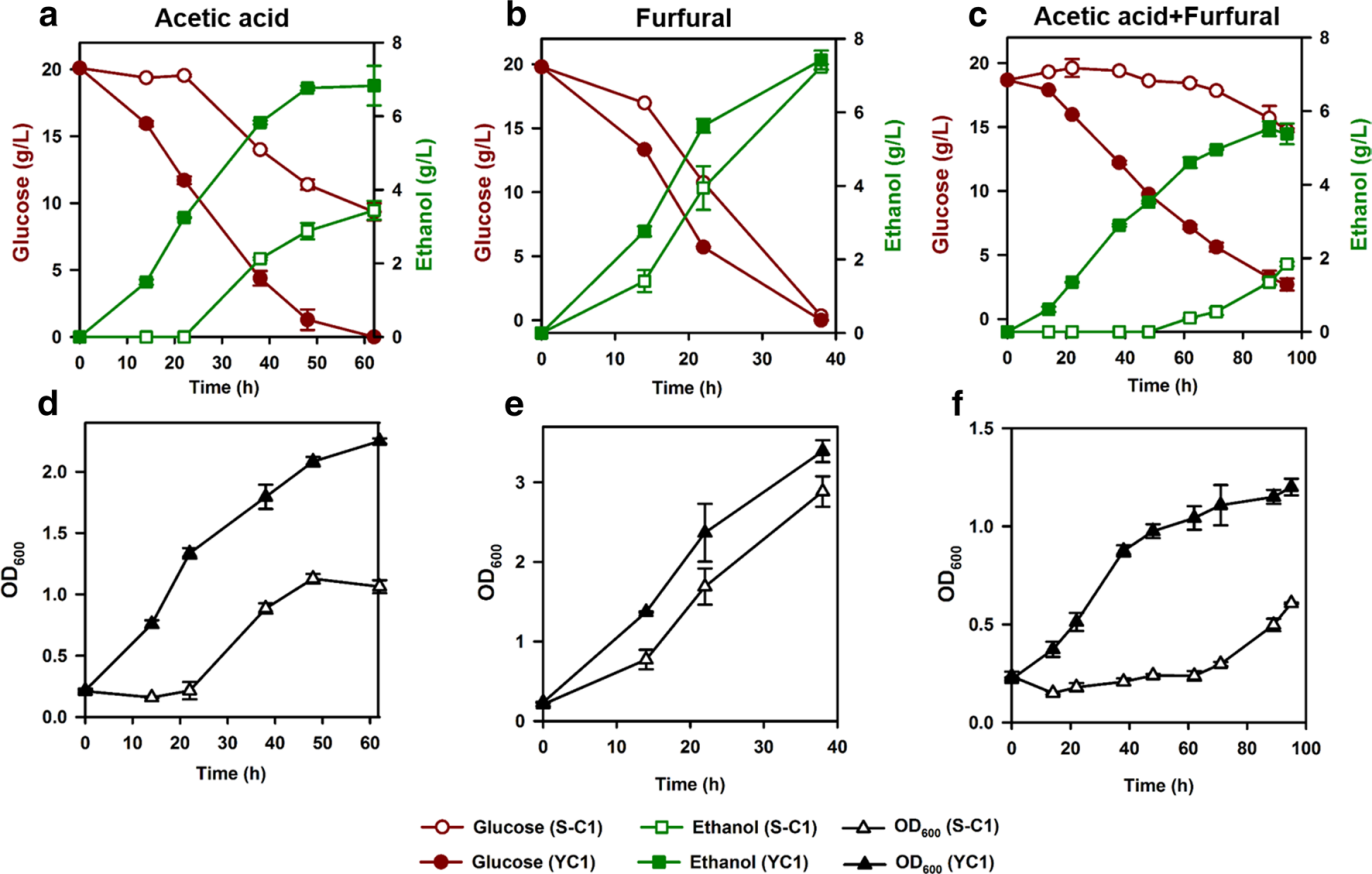
Improved fermentation by the strain YC1 compared to the control strain S-C1 in SC medium containing glucose (20 g/L) + acetic acid (2 g/L) (**a**, **d**), furfural (1.5 g/L) (**b**, **e**), or acetic acid (2 g/L) + furfural (1.5 g/L) (**c**, **f**). Results were the means of duplicate experiments; *error bars* indicating standard deviations were not visible when smaller than the symbol size

We characterized the strain and the wild-type control S-C1 in batch fermentation with the presence of acetic acid and/or furfural under oxygen-limited conditions (Fig. 1). The strain YC1 had significantly higher sugar consumption rates, ethanol productivities, and cell growth rates than the control (*t* test, *P* < 0.05) under all the inhibitor conditions, including acetic acid alone, furfural alone, and acetic acid plus furfural (Fig. 1). The fermentation performances of YC1 and S-C1 had no significant difference under the control condition without inhibitor (Additional file 1: Figure S2) suggesting that the improvement in the strain YC1 is associated with cellular response to inhibitor stress but not the ability in substrate utilization. It is worth mentioning that the strain YC1 also demonstrated significant improvement in xylose fermentation under inhibitor stress as characterized previously [57]. The presence of both acetic acid and furfural severely inhibited glucose fermentation in the control strain (Fig. 1e) compared to the conditions with acetic acid or furfural alone (Fig. 1a, b). Noticeably, the strain YC1 showed substantially improved resistance to the mixed inhibitors as well as to single inhibitors. Specifically, the ethanol productivity of the strain YC1 was 400 % higher than that of the control strain under acetic acid + furfural stress condition, while the enhancement was 270 % for acetic acid stress condition and 170 % for furfural stress condition. The results suggested that the strain YC1 had distinct inhibitor resistance mechanism compared to the wild-type strain S-C1 in response to acetic acid and/or furfural stress. The improved sugar fermentation was also demonstrated in corn stover hydrolysates, where fermentation by the control strain was severely inhibited [57].

### The transcriptional responses of the wild-type strain to different stress conditions

In order to reveal the mechanisms of the acetic acid and furfural tolerance of *S. cerevisiae*, the wild-type strain S-C1 has been examined under 4 different conditions, i.e., without stress (Blank), with acetic acid (AA), furfural (FF), or acetic acid and furfural (AA&FF). To systematically characterize the transcriptional responses of the strain S-C1 to these different stress conditions, the RNA-seq analysis was conducted correspondingly with three biological replicates. We then compared the gene expression profiles of the strain S-C1 under each of the three stress conditions (AA, FF, and AA&FF) to the control condition (Blank), respectively. From each of the comparisons, the transcripts would be identified as significantly up-/down- regulated by choosing the cut-off value (base mean ≥ 1000; padj ≤ 0.001) in DE-seq analysis package. The transcripts selected as differentially expressed were then used for gene ontology (GO) analysis.

**Fig. 2:**
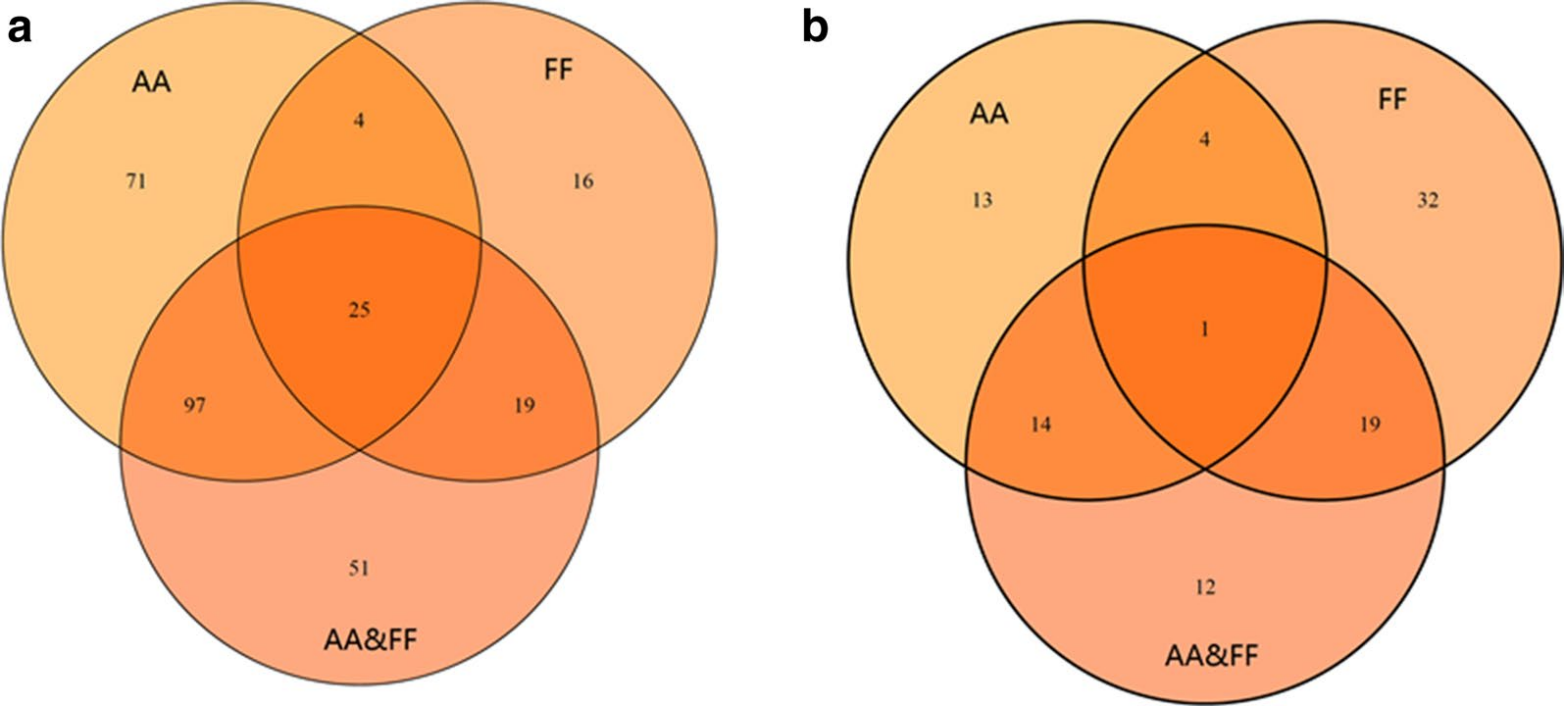
Overlapping the differentially expressed genes in the control strain S-C1 (**a**) and the strain YC1 (**b**) under different stress conditions. The gene expression profiles of growing without stress were used as the controls. *AA* growing with acetic acid; *FF* growing with furfural; *AA&FF* growing with acetic acid and furfural

In the control strain S-C1, 197 transcripts were identified to have different expression levels when growing with acetic acid, compared to those cultured under blank condition (Fig. 2 and Additional file 2: Table S1). Among them, 99 genes were up-regulated and 98 genes were down-regulated. Under furfural stress condition, 65 genes were identified to be differentially expressed, among which 20 genes were up-regulated and 45 genes were down-regulated. When the cells were cultured with both acetic acid and furfural, 192 genes had different expression levels, with 70 genes up-regulated and 122 genes down-regulated. The transcriptome shifts in response to furfural and acetic acid in *S. cerevisiae* were also reported in previous studies [66].

**Fig. 3:**
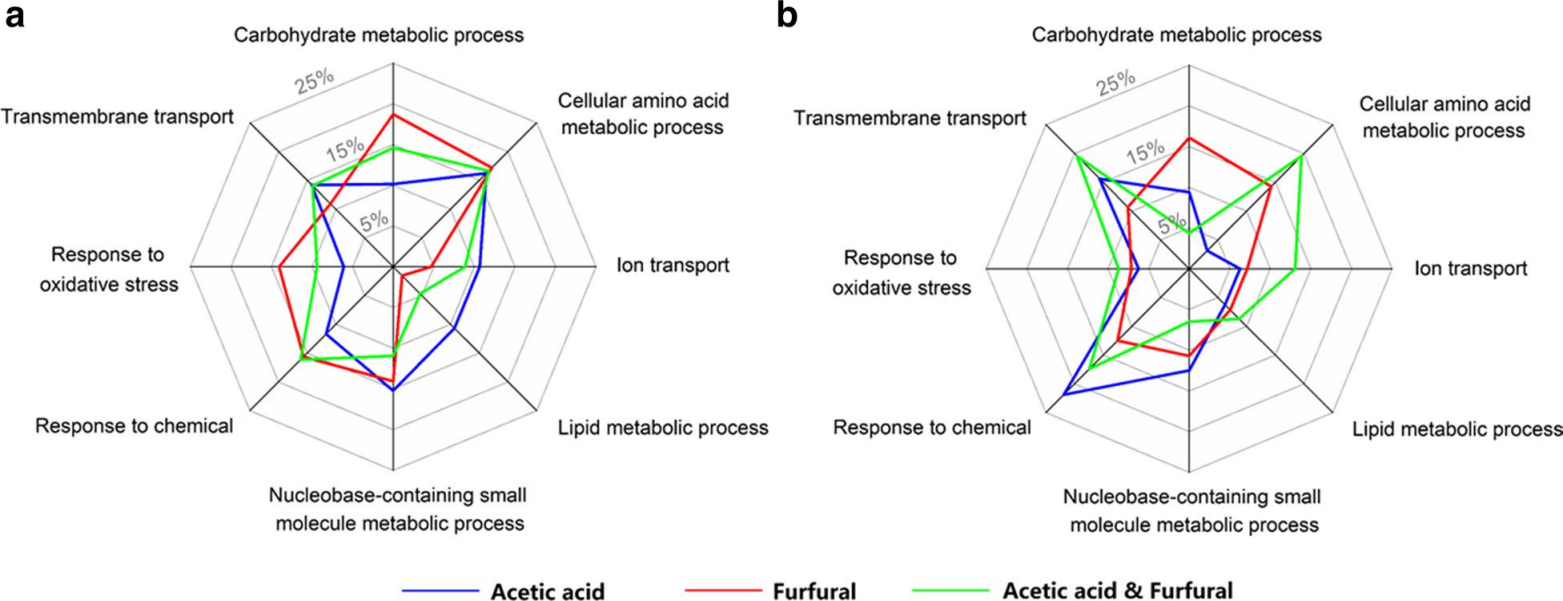
The important bioprocesses involved in response to different stress conditions in the control strain S-C1 (**a**) and the strain YC1 (**b**). Totally, eight bioprocesses were selected, since they were found to play an important role in transcriptional response to at least one stress condition. The percentage associated with each GO process was calculated as the percentage of genes involved in the corresponding GO process among the pool of genes that were significantly regulated

Based on the GO analysis, several biological processes such as cellular amino acid metabolic process (GO:0006520), nucleobase-containing small molecule metabolic process (GO:0055086), and transmembrane transport (GO:0055085) were involved as the key responses to all of the stress conditions (Fig. 3). However, the importance of these bioprocesses to respond to different inhibitors in the wild-type *S. cerevisiae* varied from condition to condition, as reflected by the different percentages of the regulated genes involved in a specific bioprocess under different stress conditions (Fig. 3). For example, lipid metabolic processes (GO:0006629) showed its significant importance in acetic acid tolerance conditions, but only one gene related to lipid metabolic processes showed different expression levels under furfural tolerance conditions (Table 3). Also, the carbohydrate metabolic bioprocess (GO:0005975) and response to oxidative stress (GO:0006979) played more important roles in furfural tolerance than that in acetic acid tolerance. The cellular amino acids metabolic process (GO:0006520) was found to be key bioprocess in all the three stress conditions, though the specific genes related to the cellular amino acids metabolic process were not the same in response to different stress conditions. As shown in Table 3, only three genes in the cellular amino acids metabolic process were always regulated regardless of the stress condition, while 17 genes, 9 genes, and 25 genes were specifically regulated in response to the stress of acetic acid, furfural, acetic acid, and furfural, respectively. Overall, the data indicated that the strain S-C1 adopted distinct endogenous genetic regulatory mechanisms to reprogram the cell metabolism in response to different stress conditions.

**Table 3:** Gene ontology analysis of transcriptional responses to different stress conditions in *S. cerevisiae* strain S-C1 (wild-type)

GO Term	Acetic acid	Furfural		Acetic acid & furfural	
Carbohydrate metabolic process (GO:0005975)	YBR001C, YBR105C, YDL055C, YDR050C, YER001W, YFL045C, YFL053W, YGL179C, YGR282C, YJL153C, *YJR096W*, YKL201C, *YLL026W, YLR286C*, YLR300W, YOL086C, YOR099W, YPL053C, YPR160W, YPR165W	YEL040W, YFR053C, YGL156W, YGR043C, YGR256W, *YJR096W*, *YLL026W*, *YLR286C*, YML100W, YMR105C, YOR120W, YPR160W			YBR001C, YBR105C, YCR005C, YDL055C, YER001W, YFL053W, YFR053C, YGL156W, YGL179C, YGR043C, YGR256W, YGR279C, YGR282C, YHR104W, YIL045W, YIL107C, YJL153C, *YJR096W*, *YLL026W*, *YLR286C*, YLR300W, YLR342W, YML070W, YML100W, YMR307W, YNL066W, YOR099W, YPL053C
Cellular amino acid metabolic process (GO:0006520)	YBL098W, YDL168W, YDL182W, YDR019C, YDR135C, *YDR502C*, YER026C, YER043C, *YER091C, YGL256W*, YGR209C, YIL074C, YJL101C, YJR010W, YJR137C, YJR139C, YKL001C, YKR080W, YLR058C, *YLR142W*, YLR180W, YLR231C, YLR303W, *YMR169C*, YMR189W, YMR300C, YNL135C, YNL160W, YOL058W, YOL086C, YOR184 W, YOR375C	YCL030C, YDR007W, *YDR502C*, *YER091C*, YGL245W, *YGL256W*, YJL200C, YJR016C, *YLR142W*, *YMR169C*, YMR250W	YCL030C, YCR005C, YDL168W, YDL182W, YDR007W, YDR135C, *YDR502C*, YDR513W, YEL046C, YER043C, *YER091C*, YFL030W, YGL009C, *YGL256W*, YGR209C, YGR267C, YIL074C, YJL101C, YJR010W, YJR137C, YJR139C, YKL001C, YKR080W, YLR058C, *YLR142W*, YLR180W, YLR303W, *YMR169C*, YMR189W, YNL160W, YOL064C, YPL091W		
Lipid metabolic process (GO:0006629)	YER026C, YER043C, YGL055W, YGR060W, YGR175C, YHR007C, YJL153C, YJL167W, YJR073C, YKL165C, YLR099C, YML008C, YNL111C, YNL169C, YNL231C, YOL151W, YOR086C, YOR317W, YPL028W, YPL095C, YPL117C	YKL150W	YER043C, YJL153C, YKL165C, YLR099C, YNL169C, YNL231C, YOL064C, YOL151W, YPL095C		
Nucleobase-containing small molecule metabolic process (GO:0055086)	YBL022C, YBL098W, *YCR021C*, YDL055C, YDL234C, YDR011W, YDR050C, YDR135C, YDR214W, YDR502C, YER043C, YER070W, YFL045C, YGR209C, YGR281W, YKR080W, YLR043C, YLR180W, YLR231C, YMR120C, YMR300C, YNL112W, YNL220W, YOL086C, YOR128C, YOR153W, YOR184W, YOR328W, YPL036 W, YPL058C	*YCR021C*, YDR502C, YGR043C, YGR256W, YKL151C, YLR249W, YMR105C, YOR128C, YPL036W	YBL022C, *YCR021C*, YDL055C, YDR011W, YDR135C, YDR214W, YDR502C, YEL039C, YER043C, YFL037W, YGR043C, YGR209C, YGR256W, YGR281W, YKR080W, YLR180W, YMR120C, YNL220W, YOR153W, YOR328W, YPL058C		
Response to chemical (GO:0042221)	YBL022C, YBR008C, YBR101C, *YCR021C*, YDL020C, YDL124W, YDR011W, YDR135C, *YDR533C*, YFL053W, YJL101C, YJR096W, YKL178C, YKR071C, YLL026W, YLR043C, YLR109W, YML116W, YNL231C, *YOR052C*, YOR153W, YOR328W, YPL026C	*YCR021C*, *YDR533C*, YFL014W, YGR088W, YJR096W, YKL150W, YLL026W, YMR250W, *YOR052C*, YOR120W	YBL022C, YBL064C, YBR008C, *YCR021C*, YDL020C, YDR011W, YDR135C, YDR513W, YDR533C, YEL047C, YFL014W, YFL037W, YFL053W, YGR088W, YHR008C, YHR104W, YJL101C, YJR096W, YKL178C, YKR066C, YKR071C, YLL026W, YML070W, YML116W, YNL231C, YOL052C-A, *YOR052C*, YOR153W, YOR328W, YPL026C, YPL091W		
Response to oxidative stress (GO:0006979)	YBL022C, *YCR021C*, YDL124W, YDR222W, *YDR533C*, YJL101C, *YJR096W*, YKR071C, YLL026W, YLR043C, YLR109W, YLR205C	*YCR021C*, *YDR533C*, YFL014W, YGR088W, *YJR096W*, YKL150W, YLL026W, YMR250W, YOR120W	YBL022C, YBL064C, *YCR021C*, YDR222W, YDR513W, *YDR533C*, YFL014W, YGR088W, YHR008C, YHR104W, YJL101C, *YJR096W*, YKR066C, YKR071C, YLL026W, YLR205C, YOL052C-A, YPL091W		
Transmembrane transport (GO:0055085)	YAL053W, YBR008C, *YBR054W*, YCL025C, *YCR021C*, YDR011W, YDR135C, *YDR345C*, YER060W, YGL255W, YGR065C, *YGR138C*, YGR260W, YGR281W, YHL036W, YHR092C, *YHR096C*, YKL175W, YKR039W, YLL028W, YLR130C, YLR237W, YML116W, YOL119C, YOR153W, YOR306C, YOR328W, YPL036W, YPL058C, YPL274W	*YBR054W*, YCL025C, *YCR021C*, *YDR345C*, YER103W, *YGR138C*, YHR094C, *YHR096C*, YPL036W	YBL075C, YBR008C, *YBR054W*, YBR069C, *YCR021C*, YDR011W, YDR046C, YDR135C, *YDR345C*, YDR497C, YER103W, YFL054C, YGL255W, *YGR138C*, YGR281W, YHR092C, *YHR096C*, YJR095W, YKL175W, YLL028W, YLR237W, YLR259C, YML116W, YOR153W, YOR328W, YPL058C, YPL274W, YPR138C, YPR156C		
Ion transport (GO: 0006811)	YAL053W, YBR054W, YCL025C, *YCR021C*, YDR135C, YGL255W, YGR065C, YGR138C, YGR142W, YGR260W, YHL036W, YKL165C, YKL175W, YKR039W, YLL028W, YLR130C, YLR214W, YML116W, YNL231C, YOR306C, YOR317W, YPL036W, YPL274W	YBR054W, YCL025C, *YCR021C*, YGR138C, YPL036W	YBR054W, YBR069C, *YCR021C*, YDR046C, YDR135C, YGL255W, YGR138C, YGR142W, YHR162W, YJR095W, YKL165C, YKL175W, YLL028W, YML116W, YMR266W, YNL231C, YPL274W, YPR138C, YPR156C		

The genes marked in italics indicate the common genes regulated in the same bioprocess (GO Term) under different stress conditions

### The transcriptional responses of inhibitor-resistant strain to different stress conditions

We next determined and compared the transcriptional responses of the inhibitor-resistant strain YC1 under different stress conditions. Basically, compared to the transcriptional profiles in the blank condition, 32 genes were identified to have different expression levels when YC1 was cultured under acetic acid stress condition, including 19 up-regulated genes and 13 down-regulated genes. Under furfural stress condition, 56 genes were identified to have different expression levels, with 40 genes up-regulated and 16 genes down-regulated. When the cells were cultured with both acetic acid and furfural, 46 genes showed different expression levels, among which 42 genes were all up-regulated and 4 genes were down-regulated (Fig. 2 and Additional file 3: Table S2). It is interesting to notice that fewer genes were differentially expressed in YC1 compared to that in S-C1, which could attribute to the activation of general stress responses by up-regulation of *WHI2*. It was found that Whi2 could interact with Msn2/Msn4 for full activation of gene expressions controlled by stress-responsive elements [67, 68]. The key biological processes involved in stress response in the strain YC1 under different inhibitor stress conditions include cellular amino acid metabolic process (GO:0006520), nucleobase-containing small molecule metabolic process (GO:0055086), and transmembrane transport (GO:0055085) (Fig. 3). However, the gene targets that were subject to be regulated in each of the bioprocess were very different. In fact, no gene was universally regulated in all of the stress conditions (Table 4).

**Table 4:** Gene ontology analysis of transcriptional responses to different stress responses in the *S. cerevisiae* strain YC1

GO Term	Acetic acid	Furfural	Acetic acid & furfural
Carbohydrate metabolic process (GO:0005975)	YLR258W, YML070W, ***YPR160W***	YDL095W, YDR001C, ***YEL040W***, YFL053W, ***YGR256W***, YKL085W, ***YML100W***, ***YMR105C***, ***YPR160W***	***YBR001C***, YKL085W
Cellular amino acid metabolic process (GO:0006520)	***YLR142W***	***YDR007*** ***W***, YDR380W, YGL184C, YJR010W, YJR137C, YKL001C, ***YLR142W***, YMR169C	***YDL168W***, ***YDR007W***, YDR035W, ***YFL030W***, YGL184C, ***YJL101C***, ***YJR010W***, ***YJR137C***, ***YMR169C***
Lipid metabolic process (GO:0006629)	YOL151W, YPL095C	YGL055W, YGL205W, ***YKL150W***, YNL169C	YGL205W, YKL150W, ***YOL151W***, ***YPL095C***
Ion transport (GO:0006811)	***YCL025C***, YML116W	***YBR054W***, YGR142W, YJR095W, YOR306C, YPL274W	YGR055W, ***YGR142W***, ***YJR095W***, YML116W, YOL130W, YOR306C
Nucleobase-containing small molecule metabolic process (GO:0055086)	***YDL234C***, ***YDR011W***, YKL173W, ***YOR153W***	YFL037W, ***YGR256W***, ***YKL151C***, ***YMR105C***, YMR120C, YNL200C	***YDR011W***, ***YGR281W***, ***YOR153W***
Response to chemical (GO:0042221)	***YBR008C***, ***YDR011W***, YDR346C, YGR234W, YML070W,***YML116W***, ***YOR153W***	YDL095W, ***YFL014W***, YFL037W, YFL053W,***YGR088W***, ***YKL150W***, YOL052C-A	***YBR008C***, ***YDR011W***, ***YDR533C***, ***YGR088W***, ***YJL101C***, YKL150W, ***YML116W***, ***YOR153W***
Response to oxidative stress (GO:0006979)	YDR346C, YGR234W	***YFL014W***, ***YGR088W***, ***YKL150W***, YOL052C-A	***YDR533C***, ***YGR088W***, ***YJL101C***, YKL150W
Transmembrane transport (GO:0055085)	***YBR008C***, ***YCL025C***, ***YDR011W***, ***YML116W***, ***YOR153W***	***YBR054W***, YCR023C, YHR092C, ***YHR096C***, YJR095W, YOR306C, YPL274W	***YBR008C***, ***YDR011W*** **, YGR055W,** ***YGR281W***, ***YJR095W***, ***YML116W***, YOL130W, ***YOR153W***, YOR306C

No common gene regulated under different stress conditions within the same bioprocess (GO Term) was found. The italic bold genes indicate the genes that were also differentially regulated in the strain S-C1 under the same conditions

Notably, the impacts of different bioprocesses on resistance to stress conditions in the resistant strain YC1 were not the same as that in the strain S-C1 (Fig. 3). For example, the response to chemicals (GO:0042221) played a more important role in resistance to acetic acid in the strain YC1 (i.e., 7 genes involved in response to chemicals were differentially expressed in a pool of totally 32 genes that were differentially expressed) than in the control strain S-C1 (i.e., 23 genes were differentially expressed in a pool of totally 197 genes that were differentially expressed), while cellular amino acids metabolic process (GO:0006520) was more important in S-C1 (i.e., 32 genes were differentially expressed in a pool of totally 197 genes that were differentially expressed in S-C1, while 1 gene was differentially expressed in a pool of totally 32 genes that were differentially expressed in YC1). Similarly, response to oxidative stress (GO:0006979) was a key biological process involved in transcriptional response to furfural in the strain S-C1, while its contribution to furfural resistance in the strain YC1 was relatively small (i.e., 9 genes were differentially expressed in a pool of totally 65 genes that were differentially expressed in S-C1, while 4 genes were differentially expressed in a pool of totally 56 genes that were differentially expressed in YC1). Under acetic acid and furfural mixed inhibitor condition, main biological processes for the stress responses in both strains included response to chemicals (GO:0042221), cellular amino acid metabolic process (GO:0006520), and transmembrane transport (GO:0055085). However, the impacts of these processes were not the same in the two strains; the transcriptional responses of the strain YC1 to mixed inhibitors were slightly more concentrated in cellular amino acid metabolic process and transmembrane transport compared to that of the strain S-C1. In general, 32 genes involved in cellular amino acid metabolic process were differentially expressed in a pool of totally 192 genes that were differentially expressed in S-C1, while 9 genes were differentially expressed in a pool of totally 46 genes that were differentially expressed in YC1. Also, 29 genes involved in transmembrane transport were differentially expressed in a pool of totally 192 genes that were differentially expressed in S-C1, while 9 genes were differentially expressed in a pool of totally 46 genes that were differentially expressed in YC1. These results indicated that the resistant strain YC1 had altered genetic regulatory networks and applied distinct molecular mechanisms (e.g., tuning the expression levels of genes involved in different biological pathways) from the wild-type strain to achieve improved stress response to acetic acid and/or furfural.

### The consensus transcriptional responses to different stress conditions

In order to find the genetic traits leading to the enhanced resistance to different inhibitor stress conditions, we compared the gene expression profiles between the resistant strain YC1 and the control strain S-C1 under each of the stress conditions. In general, 455 genes were identified to have different expression levels under the blank condition between the two yeast strains, while 536, 407, and 399 genes were identified to be differentially expressed in the strain YC1 when growing with acetic acid, furfural, acetic acid and furfural, respectively. We overlapped all of these differentially expressed genes and found 184 consensus genes that were always differentially regulated between the resistant strain YC1 and the control strain. Among the 184 consensus genes, 168 were found to be universally up-regulated in each of the stress condition, while 25 genes were universally down-regulated (Additional file 1: Figure S3, Additional file 4: Table S3). These consensus genes could most likely lead to the improved resistance to different stress conditions in the resistant strain YC1. Interestingly, we found one gene, YFL057C, demonstrated bifurcated behaviors in different stress conditions. The YFL057C gene expression was up-regulated under blank and furfural condition, but down-regulated under acetic acid and acetic acid and furfural conditions. This indicated that the effects of regulating YFL057C expression could be contradictory for yeast resistance to acetic acid and furfural. The GO analysis for these consensus genes (Table 5) indicated several key biological processes involved, including carbohydrate metabolic process (GO: 0005975), response to chemical (GO: 0042221), transmembrane transport (GO: 0055085), cellular amino acid metabolic process (GO: 0006520), and nucleobase-containing small molecule metabolic process (GO: 0055086).

**Table 5:** Gene ontology analysis of the consensus transcriptional responses by comparing gene expression profiles of the inhibitor-resistant strain YC1 and the wild-type strain S-C1 across different stress conditions

GO term (GO ID)	Genes Annotated to the GO term
Carbohydrate metabolic process (GO:0005975)	YBR001C, YBR105C, YCL018W, YCL040W, YCR005C, YDL174C, YDL193W, YER062C, YFL053W, YGL134W, YGR254W, YHR046C, YHR174W, YJR096W, YKL201C, YLL026W, YML100W, YMR135C, YMR145C, YNR001C, YOL032W, YOL059W, YOL086C, YOL136C, YOR299W
Response to chemical (GO:0042221)	YBR006W, YBR101C, YCR021C, YDL124W, YDR135C, YFL053W, YFR022W, YGR008C, YJL034W, YJL128C, YJR096W, YKL062W, YKL073W, YKL109W, YKR066C, YKR071C, YLL026W, YLR350W, YMR250W, YNL007C, YOL081W, YPL026C, YPL239W, YPR036W-A
Transmembrane transport (GO:0055085)	YAL005C, YBR287W, YCL025C, YCR021C, YCR023C, YDR046C, YDR086C, YDR135C, YDR345C, YEL024W, YER103W, YGL006W, YGR065C, YGR138C, YHL036W, YHR092C, YJL034W, YKL073W, YKL174C, YLL024C, YNL125C, YPL036W, YPR156C
Cellular amino acid metabolic process (GO:0006520)	YBR006W, YCL018W, YCR005C, YDL182W, YDR135C, YGL196W, YIR034C, YJR078W, YJR103W, YJR109C, YJR137C, YLR142W, YMR250W, YNL037C, YNL073W, YNR001C, YOL086C, YOR136W, YOR202W, YPL160W, YPR035W
Nucleobase-containing small molecule metabolic process (GO:0055086)	YCR021C, YDR135C, YDR529C, YEL021W, YEL024W, YEL041W, YER036C, YER037W, YJR078W, YJR103W, YKL073W, YMR145C, YNL088W, YNL220W, YOL059W, YOL081W, YOL086C, YOR204W, YPL036W, YPR181C

**Fig. 4:**
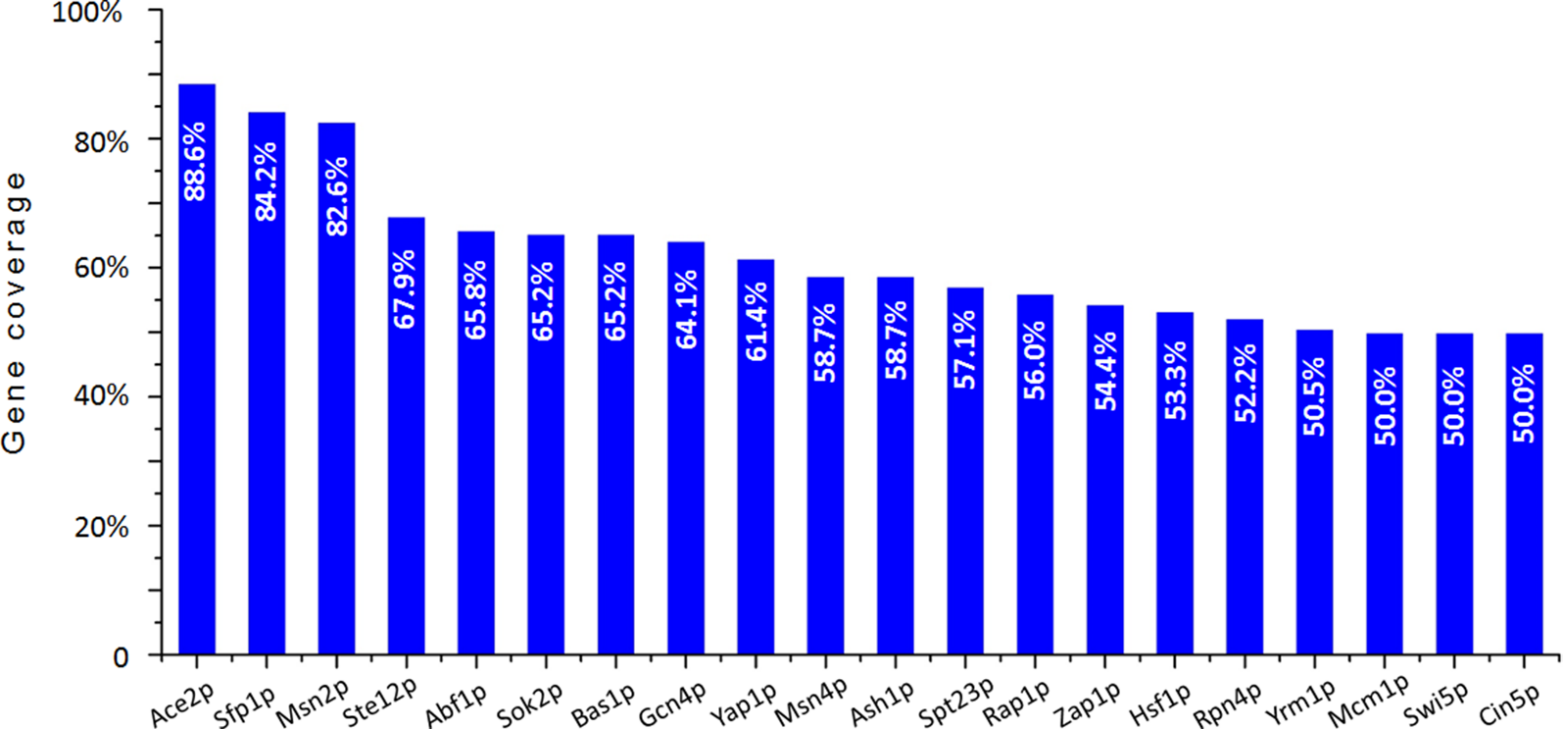
Transcription factor (TF) profiles for regulating the consensus genes involved in response to different stress conditions. The percentage of genes regulated by each of the top 20 TFs was calculated as the number of genes regulated by the TF relative to the total number of consensus genes involved in response to different stress conditions

Since the exhibition of complex genetic responses of *S. cerevisiae* to the environment was largely due to the transcription factors (TFs) that control the flow of genetic information from DNA to mRNA, we next applied a TF analysis to identify the TFs evolved in regulating the consensus genes using the YEASTRACT database. The percentage of genes a TF can regulate was defined as the ratio of the number of consensus genes the TF can regulate to the number of total consensus genes. We then generated a TF profile by choosing the top 20 candidates based on the coverage of genes they regulated (Fig. 4). Among the top four TF candidates, three of them (Ace2p, Sfp1p, and Ste12p) participate in regulating the life cycle and carbon metabolism of the yeast, while one TF (Msn2p) was found to involve in chemical responses. The most important TFs identified were Ace2p and Sfp1p, which were ranked as the top 1 and 2 TFs that are involved in regulating yeast response to acetic acid and furfural stress. Ace2p encodes a transcription factor that belongs to the C2H2 zinc finger class [69]. At the end of mitosis, Ace2p acts specifically in daughter cells to activate transcription of genes such as chitinases and glucanases that are required to destroy the septum and allow mother and daughter cells to separate after budding [69]. Sfp1p regulates the expression of nearly 10 % of yeast genes, most of which are involved in ribosome biogenesis and the regulation of cell size. Under optimal growth conditions, Sfp1p is localized to the nucleus and helps promote ribosome protein (RP) gene expression by binding to the promoters of RP genes [70]. When cells suffered nutrient limitation or chemical stress, Sfp1p is released from RP gene promoters and leaves the nucleus. Sfp1p mediates the information from stress-responsive signaling pathways to the regulation of RP gene expression and then finally bridges the physiological changes corresponding to the environment.

### Effects of overexpressing *ACE2* or *SFP1* on resistance to acetic acid and furfural

**Fig. 5:**
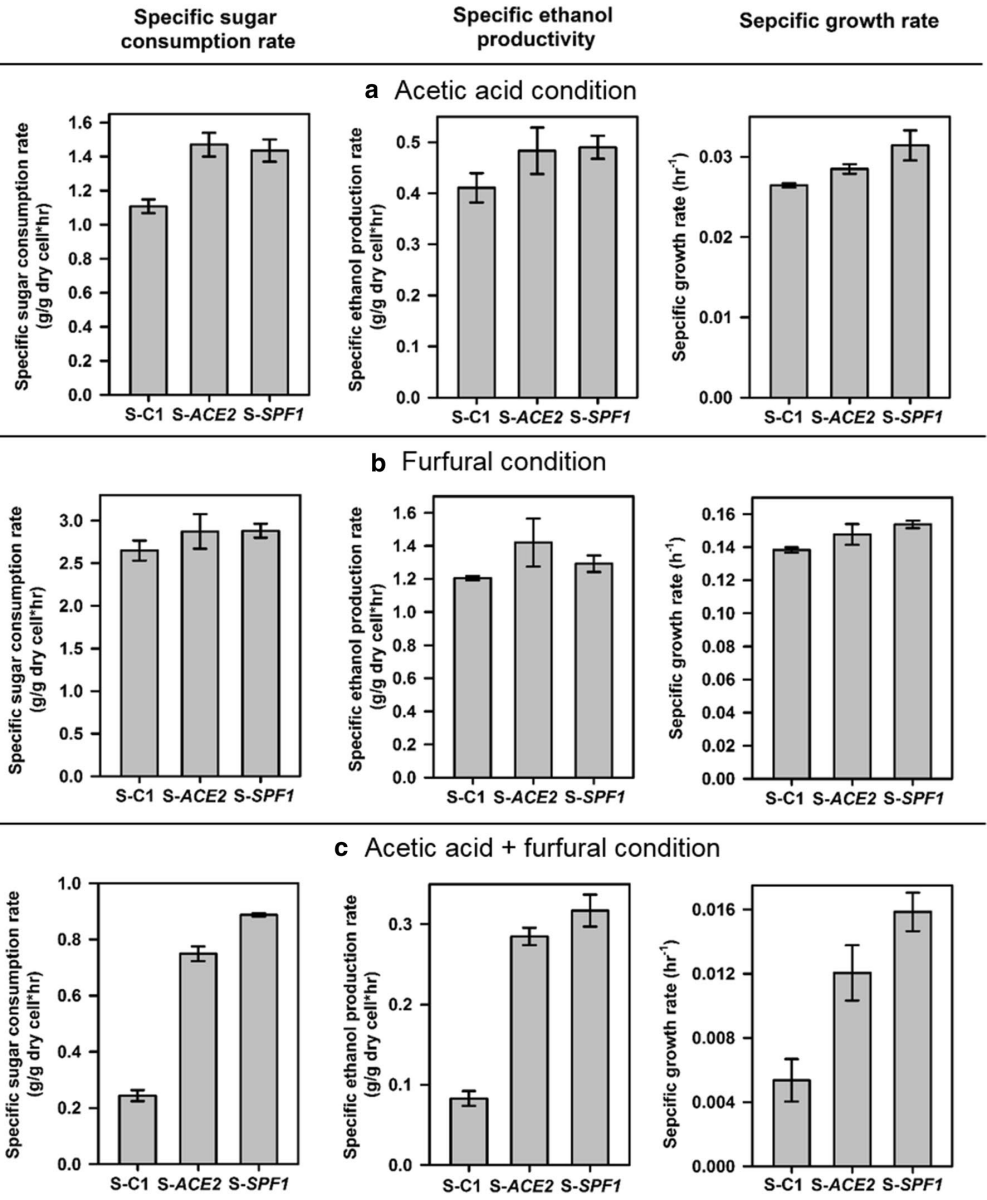
Fermentation performances of the strains S-ACE2, S-SFP1, and the control S-C1 under conditions with acetic acid (**a**), furfural (**b**), or acetic acid + furfural (**c**). *Bars* represent specific sugar consumption rates, specific ethanol productivities, and specific cell growth rates. Results were the means of duplicate experiments and *error bars* indicated standard deviations

Since the TFs identified through the TF analysis are involved in regulating consensus genes related to stress response to acetic and furfural in *S. cerevisiae*, we hypothesized that the inhibitor resistance phenotype could be possibly altered when the relevant TFs are perturbed. We chose the top two TFs Ace2p and Sfp1p and evaluated the effects of their overexpression on inhibitor resistance in *S. cerevisiae*. Two engineered yeast strains were constructed by overexpressing the *ACE2* gene or *SFP1* gene in the parent strain SR8-*trp*, respectively, yielding two new strains *S*-*ACE2* and *S*-*SFP1* (Table 1). Performances of the strains *S*-*ACE2*, *S*-*SFP1* and the control *S*-*C1* in glucose fermentation under three different inhibitor stress conditions (acetic acid alone, furfural alone, and acetic acid + furfural) were compared (Fig. 5). Overall, overexpression of *ACE2* or *SFP1* resulted in significant increase in specific sugar consumption rates, ethanol productivities, and cell growth rates in the engineered strains compared to the control under different inhibitor conditions (*t* test, *P* < 0.05). In fermentation with the presence of acetic acid and furfural, overexpression of *SFP1* improved specific ethanol productivity by nearly four times, while overexpression of *ACE2* enhanced the rate by three times. The positive effects of overexpressing *SFP1* or *ACE2* were also determined in another wild-type strain, *S. cerevisiae* CEN.PK. Specific cell growth rates in the presence of acetic acid (2.5 g/L) + furfural (1.5 g/L) stress were enhanced by 25 % (for *SFP1* overexpressing strain) and 18 % (for *ACE2* overexpressing strain) compared to the wild-type control containing backbone plasmid. Additionally, we compared the effects of Sfp1p and Ace2p to Haa1p in terms of acetic acid resistance, since Haa1p is a well-known transcription factor regulating yeast stress response to acetic acid [44, 45]. Strains overexpression of *SFP1* or *ACE2* had improved specific sugar consumption rate similar to the strain overexpressing *HAA1*, while *HAA1* overexpression elicited better cell growth under acetic acid stress (Additional file 1: Figure S4). The results suggest that the perturbation of the transcription factor Sfp1p or Ace2p could elicit alteration of genetic regulatory networks which provided protection effects against acetic acid and furfural stress in *S. cerevisiae*.

**Fig. 6:**
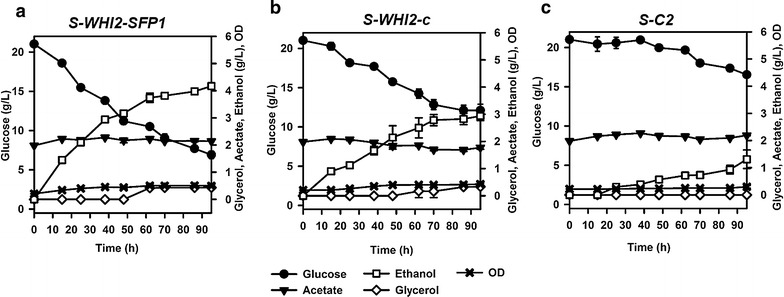
Improved fermentation by the strain S-*WHI2*-*SFP1* (**a**), compared to S-*WHI2*-*c* (**b**), and the control strain S-C2 (**c**) in SC medium containing glucose (20 g/L) + acetic acid (2 g/L) + furfural (1.5 g/L). Results were the means of duplicate experiments; *error bars* indicating standard deviations were not visible when smaller than the symbol size

In addition, since overexpression of *SFP1* had substantial effect in improving resistance to acetic acid and furfural mixture in the wild-type yeast strain, we further evaluated the effect of overexpressing *SFP1* together with *WHI2* (overexpression of which improved inhibitor resistance in the resistant strain YC1). A new group of engineered strains were constructed, including S-*WHI2*-*SPF1*, S-*WHI2*-*c*, and S-C2 (Table 1), and their fermentation performances in medium containing glucose and toxic levels of acetic acid and furfural were quantified and compared (Fig. 6). The strain S-*WHI2*-*SPF1* had the highest sugar consumption rate, ethanol productivity and cell growth rate, while the control strain only consumed less than 5 g/L glucose within the experimental time frame. Compared to the strain S-*WHI2* which overexpressed only *WHI2*, the strain S-*WHI2*-*SPF1* had 42 % increase in ethanol productivity and 20 % increase in cell growth rate, suggesting the positive effect of Sfp1p in optimizing yeast resistance to mixed fermentation inhibitors. These experimental data on the effects of Sfp1p and Ace2p indicated that the transcriptomic and bioinformatics analysis presented in this study could be a promising method framework to discover relevant TFs as genetic perturbation targets to optimize the complex phenotype such as mixed inhibitor resistance in *S. cerevisiae*.

## Discussion

In this study, the transcriptional responses of *S. cerevisiae* to fermentation inhibitor stress conditions once again confirmed that no single pathway could be found to be exclusively responsible for the resistance phenotype. Rather, global gene expressions need to be rewired to respond to the stress conditions. It is interesting to notice that the transcriptional profiles varied in response to acetic acid and furfural, which could be related to the different toxic effects of acetic acid and furfural. In general, we found that the transmembrane transport processes play pivotal roles in both resistant strain YC1 and the wild-type strain in response to acetic acid, while the carbohydrate metabolic process is crucial for stress response to furfural. Additionally, the distinct transcriptional responses to acetic acid and furfural mixture of the resistant strain versus the wild-type suggested the importance of biological processes such as transmembrane transport, cellular amino acid metabolic process, and response to chemical in regulating yeast resistance to the mixed fermentation inhibitors.

Transcription factor analysis of consensus genes that were up- or down- regulated under all of the stress conditions and blank condition in the strain YC1 versus wild-type revealed key determinants for improved yeast resistance to acetic acid and furfural. It should be noted that some differentially expressed genes from RNA-seq dataset could be just passively up- or down- regulated and may not contribute to eliciting stress responses. In order to remove the false-positives and narrow down our genetic targets related to improving the inhibitor resistance phenotype, we screened for the consensus genes across all of the cultivation conditions, followed by grouping the consensus genes by TFs that were most likely to regulate them. Among the top TFs (gene coverage above 80 %), Sfp1p emerges intensively as the transcriptional regulation of ribosomal genes in response to nutrient starvation and stress [43]. Although there is no direct evidence that whether or not Sfp1p could participate in the control of the genome-wide transcriptional response to the weak acids, it has been reported that all the molecules which could induce general nutrient limitation were also suggested to have a pro-oxidant effect in yeast cells [43], which could explain why Sfp1p stood out as an important TF in this study, since both of acetic acid and furfural have the oxidative properties inside *S. cerevisiae*. Another dominant TF, Ace2p, could activate several genes with critical roles in cell separation and control daughter cell–specific gene expression [71], which could possibly contribute to releasing the inhibition of growth rate and cell-cycle changes of *S. cerevisiae* induced by chemicals in this study. Interestingly, as discovered previously in studying stress responses of *S. cerevisiae* to HMF and furfural in xylose utilization [72], Ace2p was found to be a reporter transcription factor of the genes down-regulated after pulsing of HMF and furfural in the xylose consumption phase. This indicated a bifurcated role Ace2 could play in regulating yeast metabolism to resist different inhibitors (acetate + furfural v.s. HMF + furfural) under different sugar utilization conditions (e.g., glucose metabolism v.s. xylose metabolism).

While not tested in this study, appropriate perturbation of other top ranked TFs such as Msn2p, Msn4p, Gcn4p, Yap1p, Hsf1p, Rpn4p, and Cin5p could potentially generate beneficial genetic traits to improve yeast stress responses, since these TFs are heavily involved in regulating stress-responsive genes in yeast to resist various stress factors. For example, the participation of Msn2p in the transcriptional response to fermentation inhibitors has been documented before [42–44]. Initial characterization of the effects of overexpressing *MSN2* in the strain SR8-trp showed improved specific cell growth rate by 7 % compared to that of S-C1 under furfural (1.5 g/L) stress in synthetic complete medium, but no significant improvement was observed under acetic acid (2 g/L) stress in the experimental condition, indicating the distinct regulatory mechanism for different fermentation inhibitors. Also, the transcription factor Yap1p has been discovered previously to contribute to inhibitor tolerance in engineered *S. cerevisiae* for fermenting lignocellulosic hydrolysate [73].

This study not only identified alteration of genetic regulatory networks and related TFs through computational bioinformatics analysis, but also experimentally determined the effect of top TFs as overexpression targets in enhancing mixed inhibitor resistance in the *S. cerevisiae* strains tested in this study. The improvement brought by overexpression *SFP1* and *ACE2* (Figs. 5, 6) suggested that our approach was effective to identify transcription factor targets relevant to optimizing the target phenotype (i.e., resistance to acetic acid and furfural). Co-expressing *ACE2* and *SFP1* also significantly improved yeast resistance to acetic acid and furfural mixture compared to expressing *ACE2* or *SFP1* individually, evidenced by the enhanced specific cell growth rate (by 31 %, *P* < 0.05) and glucose consumption rate (by 19 %, *P* < 0.05), indicating there could be some synergistic effects from combined perturbation of the two TFs. On-going work is focused on characterizing the effects of the identified TFs in more detail and elucidating their functions in yeast mixed inhibitor resistance.

It should be noted that it is intrinsically complex and challenging to engineer yeast resistance to mixed fermentation inhibitors because each type of inhibitor may have distinct toxic effects and cellular stress response mechanisms [9, 10]. Our work here illustrated a novel omics-guided metabolic engineering framework where transcription factors underlying a desirable complex phenotype could be identified and perturbed for strain optimization. Specifically, the frame work includes two key components. On one hand, comparative transcriptomic analysis generates information that will be used to uncover the underlying regulatory mechanisms for the desired phenotype (e.g., inhibitor resistance) and determine key TFs involved. On the other hand, since the identified TFs regulate the genes associated with target phenotype, the TFs will serve as the promising genetic perturbation targets in metabolic engineering to improve the phenotype of interest and achieve strain optimization. While the present study conducted overexpression of the selected TFs as the initial demonstration, other perturbation strategy could also be applied such as deletion, screening of TF mutant library, and fine-tuning to determine the best possible strategy for phenotype improvement. Additionally, as the strain YC1 contains xylose-utilizing pathway, future study will determine the effects of genetic perturbation on improving fermentation of glucose and xylose (the two most abundant sugars from lignocellulosic biomass) in the presence of mixed fermentation inhibitors. Transcriptional regulatory networks could vary in xylose fermentation versus glucose fermentation in *S. cerevisiae* [74, 75], so it would be meaningful to investigate inhibitor stress response mechanisms and identify transcription factor targets eliciting improved inhibitor resistance in both sugar fermentation conditions.

Overall, in this proof-of-concept study, we confirmed the pivotal role played by TFs in stress response and demonstrated that the yeast resistance to stress factors could indeed be improved by perturbing the key TFs. Future work will systemically evaluate all the other highly ranked TFs and identify their effects on yeast stress responses to mixed fermentation inhibitors. Besides, while the present study demonstrated successful identification of novel TFs for improved yeast resistance to acetic acid and furfural, it would be meaningful to determine molecular basis and genetic targets for tolerance to phenolic compounds as well. Engineering microbial resistance to fermentation inhibitors becomes even more challenging and complex as the types of inhibitors expanded in the mixture. With the transcriptomic-guided metabolic engineering approach demonstrated in the present work, our future work will apply the method to identify new TF targets, as well as further characterize the TFs already identified in this study, for their functions in eliciting improved resistance to all the three inhibitors.

## Conclusions

In this study, we applied comparative transcriptomics analysis to advance understanding of the molecular basis for stress responses of *S. cerevisiae* to both single and mixed fermentation inhibitors of acetic acid and furfural. We identified two transcription factors, Sfp1p and Ace2p, as the pivotal regulators in *S. cerevisiae* to control the yeast resistance to acetic acid and furfural, and confirmed their positive effects on improving the inhibitor resistance via gene overexpression. To our best knowledge, it is the first time these two transcription factors were uncovered for their functions in improving yeast resistance to mixed fermentation inhibitors. The transcriptomic-guided metabolic engineering approach we demonstrated in this study could be potentially used as a strategy to improve complex phenotypes of industrial microorganisms.

## Additional files

10.1186/s13068-015-0418-5 Correlation of qPCR results and RNA-seq analysis of gene expressions (R2=0.99). **Figure S2**. Fermentation performance of the strain YC1 and the control strain S-C1in SC medium containing glucose (20 g/L) without acetic acid. Results were the means of duplicate experiments. Deviations are less than 10 %. **Figure S3**. Overlapping the differentially expressed genes by comparing the transcriptional profiles of the control strain S-C1 and the strain YC1 under different stress conditions. Blank: growing without stress; AA: growing with acetic acid; FF: growing with furfural; AA&FF: growing with acetic acid and furfural. **Figure S4**. Fermentation performances of the strains S-HAA1, S-ACE2, S-SFP1 and the control S-C1 under conditions with acetic acid. Bars represent specific sugar consumption rates and specific cell growth rates. Results were the means of duplicate experiments. Deviations are less than 15 %.
10.1186/s13068-015-0418-5 Transcriptional analysis and GO analysis of the wild-type strain to different stress conditions.
10.1186/s13068-015-0418-5 Transcriptional analysis and GO analysis of the inhibitor-resistant strain to different stress conditions.
10.1186/s13068-015-0418-5 Transcriptional analysis and GO analysis of consensus genes.

## Abbreviations

HMF: 5-hydroxymethylfurfural
RNA-seq: ribonucleic acid (RNA) sequencing
TFs: transcription factors
YP: yeast extract-peptone
SC: synthetic complete
PCR: polymerase chain reaction
qPCR: quantitative polymerase chain reaction
AA: acetic acid
FF: furfural
AA&FF: acetic acid and furfural
GO: gene ontology
RP: ribosome protein
