# Supplementary material for: Transcriptional profiling reveals molecular basis and novel genetic targets for improved resistance to multiple fermentation inhibitors in Saccharomyces cerevisiae
Source: Biotechnol Biofuels. 2016 Jan 13;9:9. doi: 10.1186/s13068-015-0418-5 (PMC4710983; doi:10.1186/s13068-015-0418-5)
Supplement: Supplementary file 1 — 10.1186/s13068-015-0418-5 Correlation of qPCR results and RNA-seq analysis of gene expressions (R2=0.99). Figure S2. Fermentation performance of the strain YC1 and the control strain S-C1in SC medium containing glucose (20 g/L) without acetic acid. Results were the means of duplicate experiments. Deviations are less than 10 %. Figure S3. Overlapping the differentially expressed genes by comparing the transcriptional profiles of the control strain S-C1 and the strain YC1 under different stress conditions. Blank: growing without stress; AA: growing with acetic acid; FF: growing with furfural; AA&FF: growing with acetic acid and furfural. Figure S4. Fermentation performances of the strains S-HAA1, S-ACE2, S-SFP1 and the control S-C1 under conditions with acetic acid. Bars represent specific sugar consumption rates and specific cell growth rates. Results were the means of duplicate experiments. Deviations are less than 15 %. [file 13068_2015_418_MOESM1_ESM.docx]

**Supplemental materials**

**Figure S1**


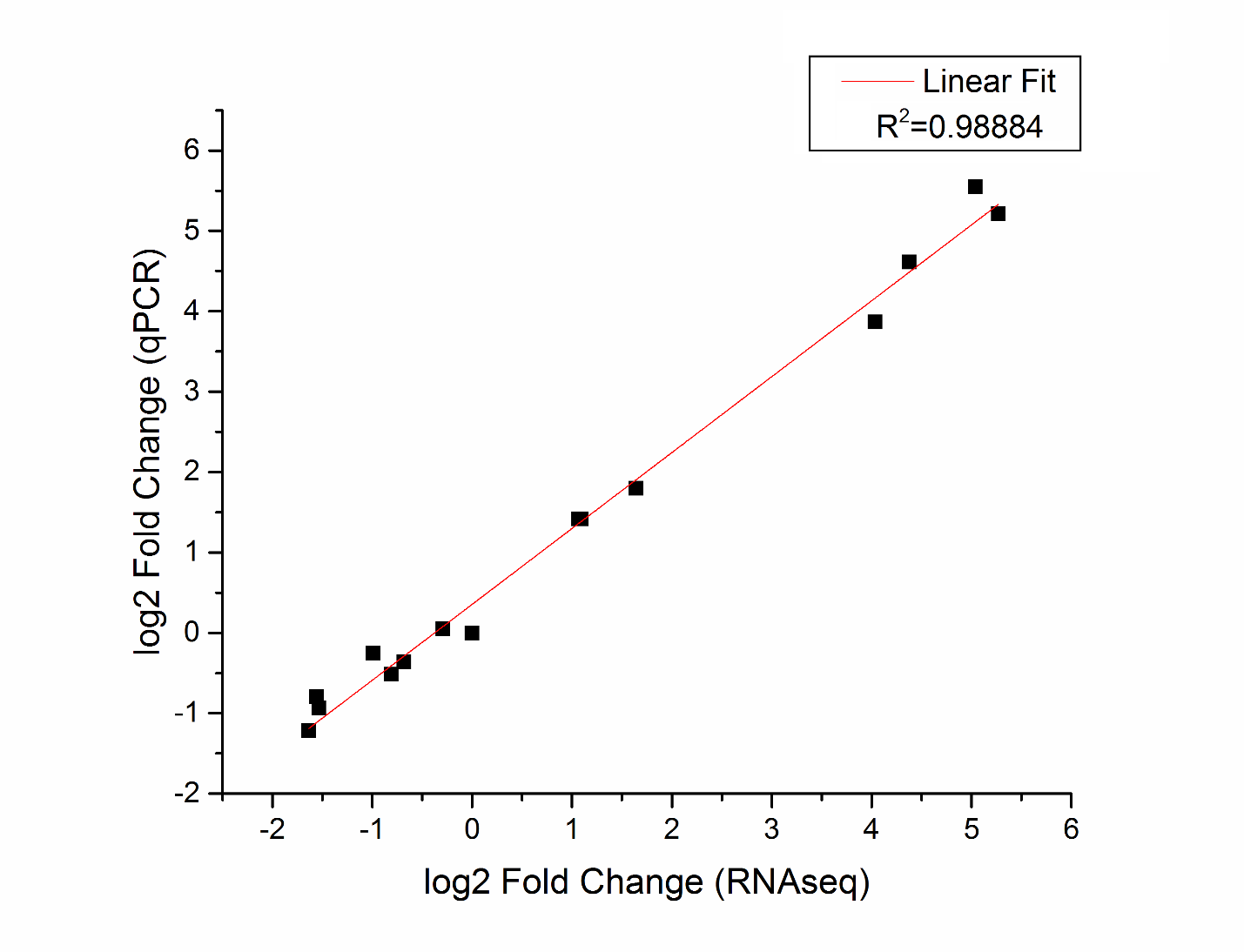


**Figure S1.** Correlation of qPCR results and RNA-seq analysis of gene expressions (R^2^=0.99)

**Figure S2**

**Figure S2.** Fermentation performance of the strain YC1 and the control strain S-C1in SC medium containing glucose (20 g/L) without acetic acid. Results were the means of duplicate experiments. Deviations are less than 10%.

**Figure S3**


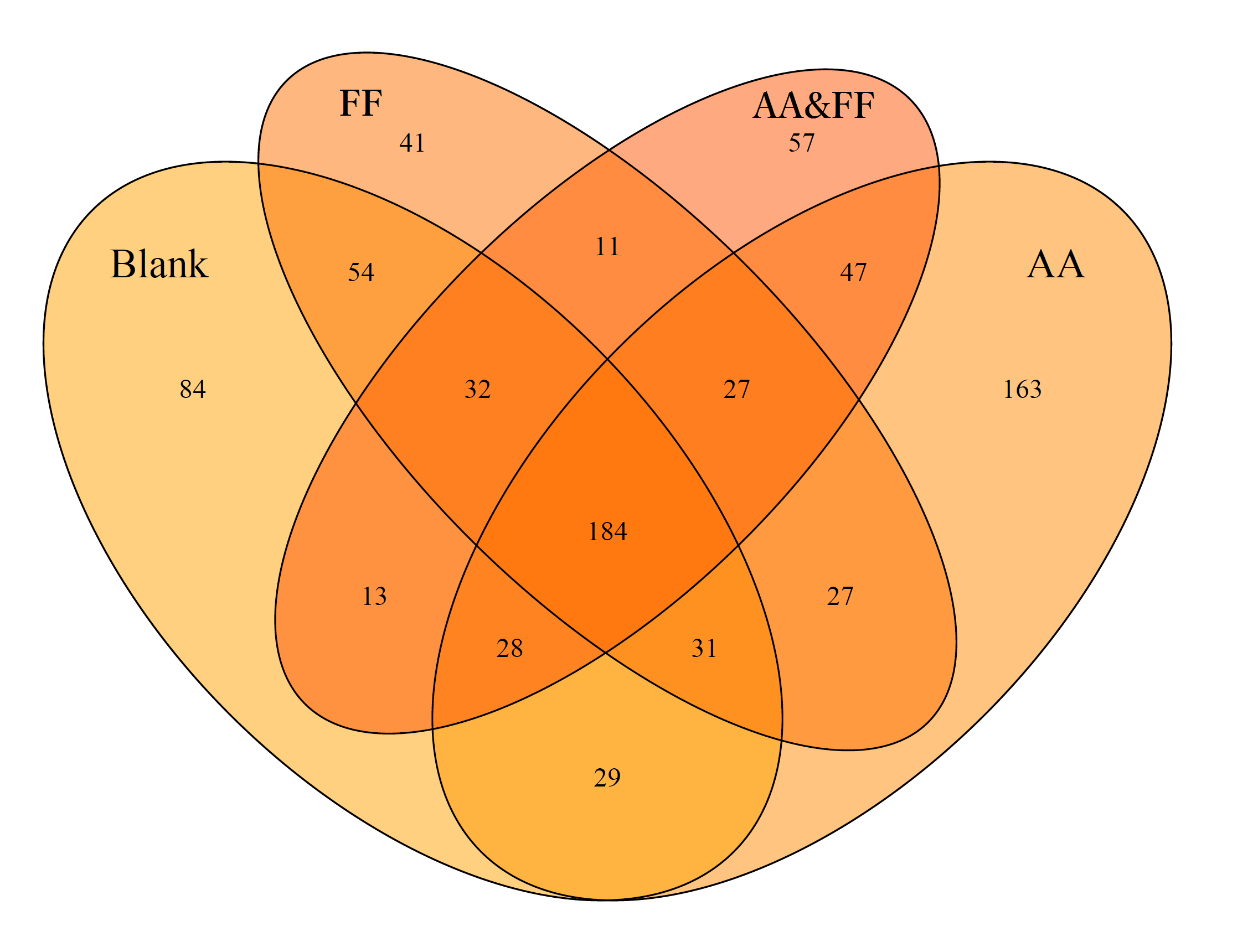


**Figure S3.** Overlapping the differentially expressed genes by comparing the transcriptional profiles of the control strain S-C1 and the strain YC1 under different stress conditions. Blank: growing without stress; AA: growing with acetic acid; FF: growing with furfural; AA&FF: growing with acetic acid and furfural.**Figure S4**

**Figure S4**. Fermentation performances of the strains S-*HAA1*, S-*ACE2*, S-*SFP1* and the control S-C1 under conditions with acetic acid. Bars represent specific sugar consumption rates and specific cell growth rates. Results were the means of duplicate experiments. Deviations are less than 15%.
